# Aqua­bis­(methacrylato-κ*O*)bis(pyridine-κ*N*)copper(II)

**DOI:** 10.1107/S1600536809012422

**Published:** 2009-04-08

**Authors:** Bin Wu, Haizhen Yao

**Affiliations:** a Department of Chemistry, Zhejiang Sci-Tech University, Hangzhou 310018, People’s Republic of China

## Abstract

In the crystal structure of the title complex, [Cu(C_4_H_5_O_2_)_2_(C_5_H_5_N)_2_(H_2_O)], the Cu^II^ cation is located on a twofold rotation axis and coordinated by two methyl­acrylate anions, two pyridine ligands and one water mol­ecule in a distorted square-pyramidal geometry. The coordinated water mol­ecule is also located on the twofold axis. In the crystal structure O—H⋯O hydrogen bonds link the mol­ecules, forming chains along the *c* axis.

## Related literature

For general background to copper complexes, see: Du *et al.* (2004 ▶); Hu *et al.* (2004 ▶); Zhu *et al.* (2007 ▶). For a related structure, see: Wu & Wang (2004 ▶).
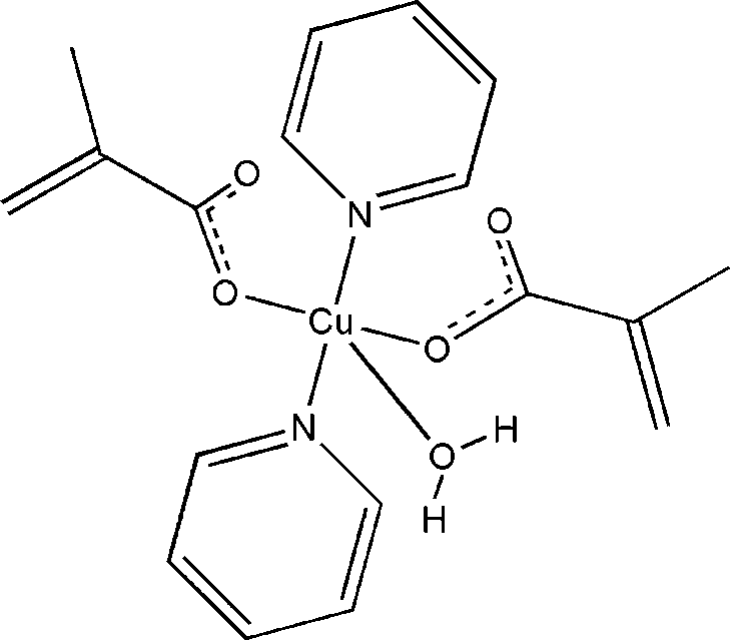


## Experimental

### 

#### Crystal data

[Cu(C_4_H_5_O_2_)_2_(C_5_H_5_N)_2_(H_2_O)]
*M* *_r_* = 409.92
Orthorhombic, 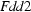

*a* = 15.619 (3) Å
*b* = 40.200 (8) Å
*c* = 6.0576 (12) Å
*V* = 3803.4 (13) Å^3^
*Z* = 8
Mo *K*α radiation
μ = 1.18 mm^−1^
*T* = 293 K
0.50 × 0.36 × 0.08 mm

#### Data collection

Rigaku R-AXIS RAPID IP diffractometer
Absorption correction: multi-scan *ABSCOR* (Higashi, 1995 ▶) *T*_min_ = 0.612, *T*_max_ = 0.913
7925 measured reflections
1808 independent reflections
1740 reflections with *I* > 2σ(*I*)
*R*_int_ = 0.027

#### Refinement

*R*[*F* ^2^ > 2σ(*F* ^2^)] = 0.021
*wR*(*F* ^2^) = 0.055
*S* = 1.09
1808 reflections
124 parameters
1 restraint
H atoms treated by a mixture of independent and constrained refinement
Δρ_max_ = 0.15 e Å^−3^
Δρ_min_ = −0.19 e Å^−3^
Absolute structure: Flack (1983 ▶), 797 Friedel pairs
Flack parameter: 0.006 (13)

### 

Data collection: *PROCESS-AUTO* (Rigaku, 1998 ▶); cell refinement: *PROCESS-AUTO*; data reduction: *CrystalStructure* (Rigaku/MSC, 2002 ▶); program(s) used to solve structure: *SHELXS97* (Sheldrick, 2008 ▶; program(s) used to refine structure: *SHELXL97* (Sheldrick, 2008 ▶); molecular graphics: *ORTEP-3 for Windows* (Farrugia, 1997 ▶); software used to prepare material for publication: *WinGX* (Farrugia, 1999 ▶).

## Supplementary Material

Crystal structure: contains datablocks global, I. DOI: 10.1107/S1600536809012422/xu2491sup1.cif

Structure factors: contains datablocks I. DOI: 10.1107/S1600536809012422/xu2491Isup2.hkl

Additional supplementary materials:  crystallographic information; 3D view; checkCIF report

## Figures and Tables

**Table 1:** Selected bond lengths (Å)

Cu—O1	2.281 (2)
Cu—O2	1.9389 (12)
Cu—N1	2.0254 (14)

**Table 2:** Hydrogen-bond geometry (Å, °)

*D*—H⋯*A*	*D*—H	H⋯*A*	*D*⋯*A*	*D*—H⋯*A*
O1—H1⋯O3^i^	0.83 (3)	1.96 (3)	2.783 (2)	178 (2)

Symmetry code: (i) 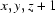.

**Fig. 1.**
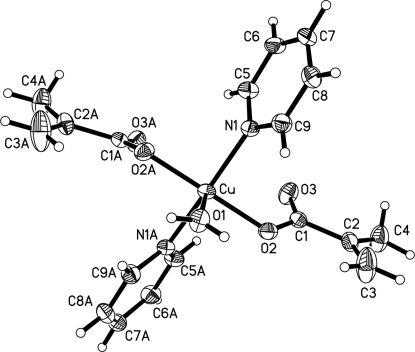
The molecular structure of the title molecule showing the atom-labelling scheme. Displacement ellipsoids are drawn at the 30% probability level for non-H atoms.

## supplementary crystallographic information

### Comment

Copper complexes with organic acids and other donor ligands exist extensively in living things, playing an important role in a vast range of chemical and biochemical catalytic systems. A series of copper-carboxylate complexes has been reported (Du *et al.*, 2004; Hu *et al.*,  2004; Zhu *et al.*,  2007).

The molecular structure is shown in Fig. 1. The Cu atom is located on a twofold axis and coordinated with two methylacrylate, two pyridine ligands and one coordinated water molecule in a distorted square-pyramidal geometry  (Table 1).

The compound is an infinite one-dimensional network structure connected by hydrogen bonds. It forms hydrogen bonds between coordination waters and carboxy group (Table 2).

The corresponding complex with one pyridine ligand has binuclear cage structural unit, two Cu atoms are bridged by four µ_2_–O,O'α-methacrylate groups, forming a cage structure (Wu *et al.*, 2004).

### Experimental

HL, CH_2_C(CH_3_)COOH, (0.5 ml, 6.0 mmol) and Cu(NO_3_)_2_.3H_2_O (240 mg, 1.0 mmol) were dissolved in 60 ml H_2_O, and the pH adjusted to 4.0 using 0.5 *M* NaOH. Two mililiters of 1.0 *M* pyridine solution were added into the mixed solution with stirring. After filtration, the filtrate was allowed to stand at room temperature and single crystals were obtained after one week.

### Refinement

Methyl H atoms were constrained to an ideal geometry with C—H distances of 0.96 Å and *U*_iso_(H) = 1.5*U*_eq_(C), but each group was allowed to rotate freely about its C—C bond. The methylene H atoms and aromatic H atoms were placed in geometrically idealized positions and constrained to ride on their parent atoms with C—H distances of 0.93 Å and *U*_iso_(H) = 1.2*U*_eq_(C).

### Crystal data

**Table:**

[Cu(C_4_H_5_O_2_)_2_(C_5_H_5_N)_2_(H_2_O)]	*F*(000) = 1704
*M**_r_* = 409.92	*D*_x_ = 1.432 Mg m^−^^3^
Orthorhombic, *F**d**d*2	Mo *K*α radiation, λ = 0.71069 Å
Hall symbol: F 2 -2d	Cell parameters from 8692 reflections
*a* = 15.619 (3) Å	θ = 3.0–27.5°
*b* = 40.200 (8) Å	µ = 1.18 mm^−^^1^
*c* = 6.0576 (12) Å	*T* = 293 K
*V* = 3803.4 (13) Å^3^	Platelet, blue
*Z* = 8	0.50 × 0.36 × 0.08 mm

### Data collection

**Table:**

Rigaku R-AXIS RAPID IP diffractometer	1808 independent reflections
Radiation source: fine-focus sealed tube	1740 reflections with *I* > 2σ(*I*)
graphite	*R*_int_ = 0.027
Detector resolution: 10.00 pixels mm^-1^	θ_max_ = 25.8°, θ_min_ = 3.3°
ω scans	*h* = −18→18
Absorption correction: multi-scan *ABSCOR* (Higashi, 1995)	*k* = −48→48
*T*_min_ = 0.612, *T*_max_ = 0.913	*l* = −7→7
7925 measured reflections	

### Refinement

**Table:**

Refinement on *F*^2^	Secondary atom site location: difference Fourier map
Least-squares matrix: full	Hydrogen site location: inferred from neighbouring sites
*R*[*F*^2^ > 2σ(*F*^2^)] = 0.021	H atoms treated by a mixture of independent and constrained refinement
*wR*(*F*^2^) = 0.055	*w* = 1/[σ^2^(*F*_o_^2^) + (0.0325*P*)^2^ + 0.6515*P*] where *P* = (*F*_o_^2^ + 2*F*_c_^2^)/3
*S* = 1.09	(Δ/σ)_max_ < 0.001
1808 reflections	Δρ_max_ = 0.15 e Å^−^^3^
124 parameters	Δρ_min_ = −0.19 e Å^−^^3^
1 restraint	Absolute structure: Flack (1983), 797 Friedel pairs
Primary atom site location: structure-invariant direct methods	Flack parameter: 0.006 (13)

### Special details

**Table:**

Experimental. Analysis: calculated C 52.74, H 5.41, N 6.83%; found C 52.61, H 5.22, N 6.69%. Spectroscopic analysis: IR (KBr, ν cm^-1^): 700, 832, 936, 1036, 1214, 1243, 1368, 1417, 1599, 1642.
Geometry. All e.s.d.'s (except the e.s.d. in the dihedral angle between two l.s. planes) are estimated using the full covariance matrix. The cell e.s.d.'s are taken into account individually in the estimation of e.s.d.'s in distances, angles and torsion angles; correlations between e.s.d.'s in cell parameters are only used when they are defined by crystal symmetry. An approximate (isotropic) treatment of cell e.s.d.'s is used for estimating e.s.d.'s involving l.s. planes.
Refinement. Refinement of *F*^2^ against ALL reflections. The weighted *R*-factor *wR* and goodness of fit *S* are based on *F*^2^, conventional *R*-factors *R* are based on *F*, with *F* set to zero for negative *F*^2^. The threshold expression of *F*^2^ > σ(*F*^2^) is used only for calculating *R*-factors(gt) *etc*. and is not relevant to the choice of reflections for refinement. *R*-factors based on *F*^2^ are statistically about twice as large as those based on *F*, and *R*- factors based on ALL data will be even larger.

### Fractional atomic coordinates and isotropic or equivalent isotropic displacement parameters (Å^2^)

**Table:**

	*x*	*y*	*z*	*U*_iso_*/*U*_eq_	
Cu	1.0000	0.0000	0.11357 (7)	0.03375 (10)	
O1	1.0000	0.0000	0.4901 (4)	0.0450 (5)	
O2	1.05574 (8)	0.04310 (3)	0.1163 (3)	0.0442 (3)	
O3	1.03022 (11)	0.05452 (4)	−0.2378 (3)	0.0529 (4)	
N1	0.88531 (9)	0.02292 (3)	0.0789 (3)	0.0361 (3)	
C1	1.05091 (11)	0.06268 (5)	−0.0494 (3)	0.0371 (4)	
C2	1.07255 (14)	0.09857 (5)	−0.0019 (4)	0.0460 (5)	
C3	1.0791 (3)	0.10857 (7)	0.2115 (5)	0.0967 (13)	
H3A	1.0922	0.1306	0.2439	0.116*	
H3B	1.0705	0.0934	0.3252	0.116*	
C4	1.0850 (2)	0.12071 (5)	−0.1829 (5)	0.0694 (7)	
H4A	1.1000	0.1423	−0.1281	0.104*	
H4B	1.0331	0.1222	−0.2672	0.104*	
H4C	1.1302	0.1125	−0.2752	0.104*	
C5	0.83858 (12)	0.01819 (5)	−0.1017 (3)	0.0431 (5)	
H5	0.8607	0.0050	−0.2143	0.052*	
C6	0.75841 (12)	0.03207 (5)	−0.1284 (8)	0.0485 (4)	
H6	0.7275	0.0286	−0.2576	0.058*	
C7	0.72506 (13)	0.05110 (5)	0.0387 (4)	0.0493 (5)	
H7	0.6705	0.0602	0.0262	0.059*	
C8	0.77349 (15)	0.05649 (5)	0.2250 (5)	0.0523 (6)	
H8	0.7525	0.0695	0.3397	0.063*	
C9	0.85367 (14)	0.04220 (4)	0.2390 (4)	0.0443 (4)	
H9	0.8868	0.0461	0.3640	0.053*	
H1	1.0098 (15)	0.0163 (6)	0.569 (6)	0.052 (8)*	

### Atomic displacement parameters (Å^2^)

**Table:**

	*U*^11^	*U*^22^	*U*^33^	*U*^12^	*U*^13^	*U*^23^
Cu	0.02640 (14)	0.04033 (15)	0.03451 (15)	0.00130 (13)	0.000	0.000
O1	0.0619 (14)	0.0422 (12)	0.0309 (11)	−0.0041 (9)	0.000	0.000
O2	0.0355 (6)	0.0454 (6)	0.0516 (8)	−0.0031 (5)	−0.0055 (7)	0.0079 (7)
O3	0.0684 (10)	0.0493 (7)	0.0410 (8)	−0.0091 (7)	0.0044 (8)	−0.0081 (7)
N1	0.0290 (7)	0.0405 (7)	0.0389 (9)	0.0003 (6)	0.0015 (7)	0.0002 (7)
C1	0.0287 (9)	0.0394 (9)	0.0432 (10)	0.0040 (7)	0.0049 (8)	−0.0027 (8)
C2	0.0534 (12)	0.0379 (9)	0.0468 (11)	0.0101 (8)	−0.0090 (10)	−0.0030 (9)
C3	0.185 (4)	0.0516 (14)	0.0534 (16)	0.0172 (18)	−0.027 (2)	−0.0070 (12)
C4	0.109 (2)	0.0436 (10)	0.0552 (16)	−0.0013 (12)	−0.0059 (15)	0.0058 (11)
C5	0.0357 (9)	0.0541 (10)	0.0394 (12)	0.0002 (8)	−0.0002 (8)	−0.0046 (8)
C6	0.0362 (9)	0.0556 (10)	0.0538 (11)	−0.0010 (8)	−0.0128 (11)	0.0031 (17)
C7	0.0319 (9)	0.0450 (10)	0.0711 (15)	0.0064 (8)	−0.0002 (10)	0.0046 (10)
C8	0.0475 (12)	0.0461 (11)	0.0634 (14)	0.0101 (9)	0.0061 (11)	−0.0088 (11)
C9	0.0419 (11)	0.0457 (9)	0.0453 (11)	0.0022 (8)	−0.0027 (9)	−0.0038 (9)

### Geometric parameters (Å, °)

**Table:**

Cu—O1	2.281 (2)	C3—H3B	0.9300
Cu—O2	1.9389 (12)	C4—H4A	0.9600
Cu—O2^i^	1.9391 (12)	C4—H4B	0.9600
Cu—N1	2.0254 (14)	C4—H4C	0.9600
Cu—N1^i^	2.0254 (14)	C5—C6	1.380 (3)
O1—H1	0.82 (3)	C5—H5	0.9300
O2—C1	1.278 (2)	C6—C7	1.371 (5)
O3—C1	1.230 (3)	C6—H6	0.9300
N1—C5	1.329 (3)	C7—C8	1.376 (4)
N1—C9	1.336 (3)	C7—H7	0.9300
C1—C2	1.510 (3)	C8—C9	1.380 (3)
C2—C3	1.357 (4)	C8—H8	0.9300
C2—C4	1.425 (3)	C9—H9	0.9300
C3—H3A	0.9300		
			
O2—Cu—O2^i^	179.03 (10)	H3A—C3—H3B	120.0
O2—Cu—N1	89.51 (5)	C2—C4—H4A	109.5
O2^i^—Cu—N1	90.59 (5)	C2—C4—H4B	109.5
O2—Cu—N1^i^	90.59 (5)	H4A—C4—H4B	109.5
O2^i^—Cu—N1^i^	89.51 (5)	C2—C4—H4C	109.5
N1—Cu—N1^i^	168.08 (10)	H4A—C4—H4C	109.5
O2—Cu—O1	89.51 (5)	H4B—C4—H4C	109.5
O2^i^—Cu—O1	89.51 (5)	N1—C5—C6	122.5 (3)
N1—Cu—O1	95.96 (5)	N1—C5—H5	118.8
N1^i^—Cu—O1	95.96 (5)	C6—C5—H5	118.8
Cu—O1—H1	126 (2)	C7—C6—C5	118.9 (3)
C1—O2—Cu	121.15 (13)	C7—C6—H6	120.5
C5—N1—C9	118.52 (16)	C5—C6—H6	120.5
C5—N1—Cu	120.41 (13)	C6—C7—C8	119.0 (2)
C9—N1—Cu	121.03 (14)	C6—C7—H7	120.5
O3—C1—O2	125.43 (17)	C8—C7—H7	120.5
O3—C1—C2	119.36 (18)	C7—C8—C9	118.9 (2)
O2—C1—C2	115.21 (18)	C7—C8—H8	120.5
C3—C2—C4	122.5 (2)	C9—C8—H8	120.5
C3—C2—C1	118.8 (2)	N1—C9—C8	122.1 (2)
C4—C2—C1	118.7 (2)	N1—C9—H9	118.9
C2—C3—H3A	120.0	C8—C9—H9	118.9
C2—C3—H3B	120.0		

Symmetry codes: (i) −*x*+2, −*y*, *z*.

### Hydrogen-bond geometry (Å, °)

**Table:**

*D*—H···*A*	*D*—H	H···*A*	*D*···*A*	*D*—H···*A*
O1—H1···O3^ii^	0.83 (3)	1.96 (3)	2.783 (2)	178 (2)

Symmetry codes: (ii) *x*, *y*, *z*+1.
